# Expectations and behaviour of older adults with neurological disorders regarding general practitioner consultations: an observational study

**DOI:** 10.1186/s12877-021-02469-3

**Published:** 2021-09-25

**Authors:** Aline Schönenberg, Ulrike Teschner, Tino Prell

**Affiliations:** 1 Department of Neurology, Jena University Hospital, Am Klinikum 1, 07747 Jena, Germany; 2 Center for Healthy Ageing, Jena University Hospital, 07747 Jena, Germany

**Keywords:** Patient GP relationship, Patient expectations, Patient behaviour, Adherence, Patient doctor relationship, Geriatric patients

## Abstract

**Background:**

Patients’ relationship with their GPs is linked to adherence, patient behaviour and satisfaction with healthcare. Several factors pertaining to this relationship have already been identified, however expectations and preferences vary depending on age and diagnosis. Chronically ill elderly patients constitute a group of patients with specific needs that are not yet understood.

**Methods:**

For this observational study, 100 (44 female, mean age 72.72 + − 8.28 years) patients were interviewed. Multiple linear or binary logistic regression as well as analysis of variance was used to understand the link between factors pertaining to GP relationship and patient behaviour, and principal component analysis was performed to understand the underlying structure of patients’ needs.

**Results:**

Patients attribute high importance to their GP’s opinion of them. On average, what the GP thinks about the patients is almost as important as what their partners think. Patients primarily want to be perceived as engaged, friendly and respected individuals, and it is important for patients to be liked by their GP. This importance is linked to active preparation; 65% of the patients prepared actively for GP consultations. Expectations regarding GP consultations can be split into two components: a medical aspect with a subfactor concerning emotional support, and a social component. Prominent factors influencing the relationship are the possibility to talk about emotions and mental well-being, trust, and GP competency. Satisfaction and trust were mainly linked to medical competency. Being able to show emotions or talk about mental well-being enhances perceived GP competence, satisfaction, and active patient preparation. However, a focus on the social component such as frequent talking about private topics reduces both perceived GP competency as well as active patient preparation.

**Conclusion:**

Older patients take GP consultations seriously, and their expectations regarding GP consultations focus on medical competence and care as well as empathetic listening and understanding. Older persons seek a deeper connection to their GPs and are willing to be active and cooperative. As the patient–GP relationship influences health outcomes, treatment of older patients should be adjusted to enable this active participation.

**Supplementary Information:**

The online version contains supplementary material available at 10.1186/s12877-021-02469-3.

## Introduction

The relationship between patients and their doctors, especially their general practitioners (GPs), is a key factor in healthcare. Prior to any diagnoses and treatment, communication between patients and GPs is essential to identify the relevant problems, risks and resources. To arrive at a shared decision, the relationship between GP and patient must be stable and functional, as it contributes not only to medical decisions but also to psychosocial well-being and care [1–5]. Many studies have shown that the relationship between patient and GP influences both the quality of medical care and the patient’s own behaviour [6–8]. In particular, previous research has shown that good communication between GP and patient significantly influences adherence [4, 9, 10] and may even decrease treatment avoidance [11], which is especially relevant as up to 50% of patients in general [12] and more than 80% of neuro-geriatric patients show nonadherence to medication [13, 14]. However, some patients report being unable to express all their needs during consultations [15], which is particularly problematic since GPs show difficulties evaluating what their patients need [16, 17]. Patients are more likely to express their wishes when they trust their GPs and when they deliberately prepared for consultations [18], indicating that a satisfying GP consultation depends on both GP and patient factors and requires active patient participation [19].

Overall, patient expectations regarding GP consultations have been described to encompass both medical and social aspects [20], and several factors relating to the patient–GP relationship have been identified, such as trust and GP competence, communication, continuity of care and personal aspects [6, 19, 21, 22]. However, it is known that expectations and needs regarding GP consultations are complex and vary between patients depending on their age and diagnosis [15, 23, 24], and studies have suggested that age and chronicity of illnesses in particular are factors related to different preferences during consultations [25, 26].

Generally, persons of advanced age constitute a particularly vulnerable population prone to experience social losses, a declining mental health, and multiple physical health problems [2, 3]. Indeed, older adults have more medication prescriptions and GP consultations than any other patient group [4, 5]. In the face of the social, mental and physical challenges of aging, this frequent GP contact becomes a central resource of social interaction, encouragement and support. Especially in this particularly frail and emotionally vulnerable elderly population, healthcare outcomes do not only depend on the medical treatment of physical health problems, but also on psychosocial care [2].

Despite this relevance, a recent review suggested that although older patients highly value a trusting relationship with their GPs, their needs are not yet fully understood and incorporated in healthcare [27]. Instead, research regarding the relationship between GPs and patients has focused either on highly vulnerable patients, such as in the field of oncology [28, 29], or on a more heterogeneous patient population encompassing a large age span with an overall view on younger patients [6, 9, 11, 30, 31]. With regard to elderly patients, some of the previous studies differed in methodology [32] or focused on individual variables [23, 33] rather than assessing the patient–GP relationship using a multitude of factors. Furthermore, several studies have focused on either difficult patients [34–36] or the contribution of the GP alone [37, 38].

This lack of awareness of the elderly patients’ needs is especially problematic since they are in such frequent contact with their doctors, making GP consultations both a necessary and valuable tool for improving adherence and health outcomes [39]. Some previous studies suggest that the preferences and needs of older and younger patients differ, especially with regard to decision making, communication style and information seeking [26, 31, 40, 41]. A particular focus of older patients lies on the psychosocial care in the GP relationship, highlighting the need to move away from the classical biomedical to a biopsychosocial interaction pattern that includes emotional support, with the authors even going as far as suggesting a therapeutic role of the GP for elderly patients [2]. Older adults focus on psychosocial aspects of medical encounters more so than younger patients do, showing specific needs for emotional support [42, 43] and a GP who takes the time to listen to them as an individual person, taking into account their personal experiences [2, 44]. This is especially relevant as GPs are often not only the first, but also the only source of professional mental health support for elderly persons [45]. Discussions about psychosocial topics and mental well-being are fruitful for patient satisfaction and better health outcomes, since this particular patient group of older persons faces a multitude of psychosocial challenges [2, 46]. Feeling rushed and sensing that their GP is not interested, on the contrary, hinders older patients from reporting important psychosocial problems [47, 48]. Additionally, the elderly population is used to a paternalistic model of patient-GP-relationship which does not incorporate psychosocial care. Thus, elderly patients may not allow themselves to speak freely about important topics such as mental well-being, both because they want to be perceived as a “good patient” and because they are not used to this form of care from their GPs [45]. This is all the more critical, as research suggests that as many as half of the symptoms elderly persons experience are not sufficiently discussed with the respective GPs, oftentimes because of a focus on the multitude of physical-health related medical symptoms which can be challenging enough for older patients to remember [49]. Therefore, in order to facilitate open communication about all relevant aspects of aging, and to enable active participation, an encouraging GP relationship is needed in which patients are motivated to convey their experiences and needs [50].

However, variables identified thus far do not seem to be sufficient to explain older patients’ needs and preferences in terms of GP consultations [27, 33]. This may, in part, be due to the common misconceptions that older adults are unable to comprehend and handle their healthcare because of cognitive decline and overall frailty [1].

To recognize the specific needs of patients with neurogeriatric conditions, it is important to understand their expectations and experiences by focusing on the patients’ perspective in view of a multitude of potential factors. The GP relationship is intricately linked to patient behaviour, medication adherence and health outcomes, making it a valuable factor in healthcare. Due to this impact of the relationship, we aimed to explore the association among relationship-related factors, patient expectations, and self-reported patient behaviour. In particular, we wanted to find out which aspects of the GP relationship are important for this specific group of older adults, and how they relate to patient behaviour, especially preparation for consultation. As both medical and psychosocial care have been named as important factors for patient satisfaction with GP consultations, we aimed to disentangle the role of medical care, mental well-being, and overall social interaction. To further explore the motivation and expectations of patients, we wanted to analyse interpersonal factors such as the role of the GP and the way patients want to be perceived to understand their wishes, needs and motivations. As there is the common misconception of “good” and “bad” patients and especially older adults tend to be devote and passive due to social norms [34, 35, 45], we explicitly aimed to understand how patients wish to be perceived by their GPs to understand how these concepts relate to self-reported behaviour and relationship factors.

A previous study reported a discrepancy between quantitative and qualitative, patient-centered approaches [20]. Therefore, this study included additional exploratory items that patients responded freely to. By doing so, we gained deeper insights not only into the patients’ needs but also into self-reported GP-related patient behavior, such as frequency of consultations, preparation or appointment cancelling, and factors that motivate or prevent patients from taking on an active role in their healthcare.

## Methods

### Participants and assessments

An exploratory, observational cross-sectional study was conducted from mid-September 2020 to November 2020 on the neurology wards of the Jena university hospital. The local ethics committee of the Jena University Hospital approved this study, and all patients provided written informed consent. Hospitalized patients aged 60 years or older with neurological diagnoses were consecutively recruited from the neurological ward, and data were collected by trained staff during face-to-face interviews. The exclusion criteria were acute illness or other circumstances leading to the inability to fill out a questionnaire, dementia, and delirium. All patients fulfilling the criteria were approached (*n* = 115), however, some patients did not want to participate (*n* = 3) or were physically unable to participate in the study (*n* = 5). Additionally, some patients were missed due to short duration of stay, early dismissal or scheduling conflicts (*n* = 3). Across those months, a total of 104 patients were interviewed until saturation was reached for the exploratory open items. Four patients were excluded from the analysis because of severe depression and acute suicidal thoughts. The datasets of the remaining 100 patients were analysed.

Interviews were conducted by psychologically trained research staff rather than doctors or nurses to ensure that patients felt comfortable speaking openly about their experiences. Each interview took place in the patients’ own hospital room rather than an office or laboratory, and was carried out in a comfortable conversation to ensure minimal response bias despite the clinical setting. No other hospital staffs were present during the interviews.

The following demographic data were collected: age, gender, family status, living situation (alone, with partner, or other), level of education (high: German Abitur or university; middle: German Realschule or General Certificate of Secondary Education; low: German Hauptschule; or no school) and employment status.

Additionally, the Patient Health Questionnaire-9 (PHQ-9) was used to assess depressive symptoms. The PHQ-9 items are based on the diagnostic criteria for depression of the Diagnostic and Statistical Manual of Mental Disorders, Fourth Edition (DSM-IV), which are assessed via self-report using a 4-point scale ranging from *0* (*not at all*) to *3* (*nearly every day*). Total scores of 10, 15 and 20 represent cut-off points for mild, moderate and severe depression, respectively [51]. Despite the clinical setting, the PHQ-9 was used strictly for scientific screening-purposes only to gauge patients’ mood as a control variable in the analysis and does not reflect a clinical diagnosis.

As most measures used in clinical research are not patient-centred and questionnaires with predetermined answers may restrict the patients’ options to express their experiences [52], the questionnaire used contained both quantitative and exploratory questions to support the quantitative data. This gave patients the opportunity to elaborate on their needs and experiences in open questions whenever they felt necessary. Answers to exploratory questions were recorded by the study staff on prepared papers during the face-to-face interviews. The questionnaire was tested on a pilot sample composed of 20 patients to assess its applicability.

The final questionnaire used consisted of 28 items divided into four categories. A translation of the full questionnaire is provided in the Supplementary Materials (Supplement Table S1). The first category consisted of nine items regarding GP-related patient behaviour. Patients’ preparation for GP consultations was evaluated using a dichotomous yes/no question and an additional exploratory item to assess any special individual preparations patients might make before consultation. Furthermore, questions about punctuality, frequency of GP consultations, additional preventive check-ups and cancellation of appointments were included. The second category consisted of 13 items assessing the factors associated with the relationship between patients and GPs (relationship factors). The variables satisfaction with GP (Satisfaction), perceived GP competency (Competency), trust in GP (Trust), showing emotions in front of GP (Showing Emotion), talking about private topics (Private Topics), GP asking about mental well-being (Well-Being), GP taking time to listen and answer questions (Time to Answer) and GP asking private questions (Private Questions) were measured using a 6-point Likert scale ranging from 6 (*always*) to 1 (*never*). The other items were open-ended and explored the tone of communication between patients and GPs, the reasons for choosing this particular GP and the duration of treatment in years. The third category explored the patients’ expectations for GP consultations. The final category assessed the importance of GPs’ perception of the patients using visual-analogue scales and open questions. Topics covered were the importance of the partner’s, colleagues’, friends’, and GP’s perceptions of the patient on a scale from 0 to 100; the patients’ thoughts on how their GP currently perceives them (Current Perception); and how they would ideally like to be perceived by their GPs (Desired Perception).

### Statistical analysis

Analysis of the quantitative data was performed using Statistical Package for the Social Sciences (SPSS 27.0; Armonk, NY, USA). In the first step, descriptive statistics such as mean, standard deviation (SD) and median were utilised to describe the available data. Categorical variables are presented as numbers or percentages. Before analysis, normal distribution was assessed using the Shapiro–Wilk test, and multicollinearity was ruled out using Spearman’s correlation for non-normally distributed data. Outliers were analysed using Cook’s Distance, and the Durbin–Watson test was used as a measure for autocorrelation.

Demographic data including age, sex, diagnosis, education level, marital status, living situation and total PHQ-9 score were included as control variables (patient-related factors). Relationship-related factors were included as independent variables.

Dependent variables were analysed using either multiple linear regression with backward selection or logistic regression with backward selection for binary variables. Significance levels for variables entering and being removed from the model were set at 0.05 and 0.01, respectively. Principal component analysis was performed to understand the underlying structure of the available data. For analysis of variance, we used the Mann–Whitney U test or Chi-square test and Fisher’s exact test to compare variables.

Significance level was set at 0.05 for all analyses and tests were applied two-sided.

The exploratory items were noted by trained staff during face-to-face interview and, if necessary, coded and sorted into categories by one of the researchers (AS). The patients’ answers were then re-categorised by two additional independent raters; inter-rater reliability was calculated using Fleiss Kappa for the three raters. All exploratory items are reported in terms of category quantity or percentages with the aim to further substantiate the quantitative data and gather a deeper understanding of the patients’ experiences.

## Results

### Description of the cohort

The final sample included 56 male (56%) and 44 female (44%) patients with a mean age of 72.72 years (SD = 8.28 years). Most patients were married, had high or medium education levels and were pensioned. According to the PHQ-9, 54 patients (54%) showed no signs of depression, 33 (33%) had mild depression and 13 (13%) had moderate to severe depression. On average, the patients reported to consult their GPs 1.5 times per quarter (M = 1.47; SD = 1.2). The patients had been with the same GP between 1 and 40 years, with an average treatment duration of 14 years. Detailed clinical and demographic characteristics are shown in Table 1. Most patients took appointments with their GPs seriously, with 79% of the patients claiming to never cancel GP consultations. Further exploratory GP-related patient behaviour, such as reasons for GP choice or use of preventive check-ups, is described in the Supplement Table S2.

**Table 1:** Clinical and demographic characteristics

			***N*** **= 100**	
Gender	Female		44	
	Male		56	
Marital Status	Married		70	
	Widowed/divorced		26	
	Single		4	
Living Situation	Alone		21	
	With others		73	
Education	Low		26	
	Middle		37	
	High		37	
Employment	Unemployed		4	
	Full-time		9	
	Part-time		2	
	Pensioned		85	
Diagnosis	Cerebrovascular disorder		39	
	Parkinson’s disease		18	
	Neuromuscular disorders		24	
	Other		19	
	**M**	**SD**	**MD**	**IQR**
Age (Years)	72.72	8.28	73.50	12
Total PHQ-9 score	4.78	4.18	4	6
Frequency of quarterly GP consultation	1.47	1.2	1	1.5
Duration of treatment by the doctor (years)	14.14	10.74	10	15

*M* Mean, *SD* Standard deviation, *MD* Median, *IQR* Interquartile range

### Patient–GP relationship and related factors

Most patients reported medical expectations regarding GP consultations, such as prescriptions (*n* = 86) and health screenings (*n* = 84). On top of medical expectations patients also mentioned a social aspect to GP consultation (*n* = 25), highlighting the importance of having a GP who listens to patients’ concerns, answers their questions and gives advice (detailed in Supplement Table S2).

**Fig. 1:**
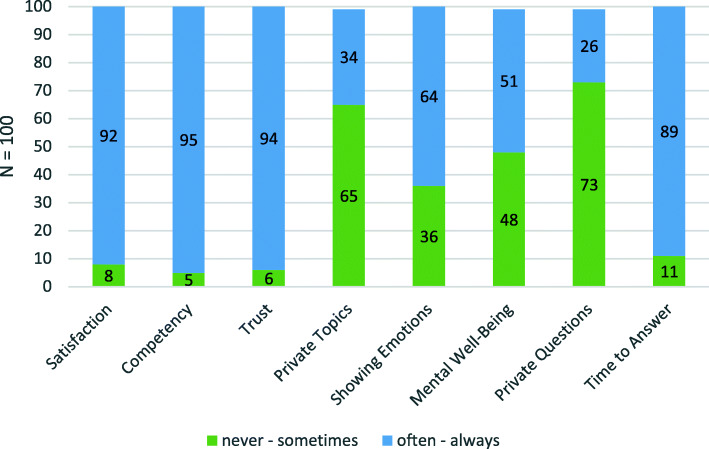
Description of relationship-related factors

Descriptive analysis revealed that most patients are highly satisfied with and trust their GPs (Fig. 1). Almost all patients perceive their GPs as competent, and most GPs seem to take enough time to answer their patients’ questions. However, the data showed a split in the cohort with regard to GPs asking about mental well-being. Approximately three-quarters of the patients rarely spoke about private topics with their GPs, and similarly most GPs did not ask private questions. With regard to showing emotions in front of GPs, approximately three-quarters of the patients reported doing so regularly.

For a more detailed understanding of the data, linear regressions with backward selection were performed for the variables Competency, Satisfaction and Trust (Table 2). This analysis revealed that patients who were satisfied with (ß = 0.248; *p* < 0.001) and trusted their GPs (ß = 0.735; *p* < 0.001), perceived their GPs as more competent (F (3, 94) = 25.982, < 0.001, corrected *R*^2^ = 0.872). However, talking about Private Topics reduced perceived Competency (ß = − 0.073; *p* = 0.052). Patients who could show their emotions (ß = 0. 037; *p* = 0.022), perceived their GP as competent (ß = 0.671; *p* < 0.001), and had a GP who asked about their mental well-being (ß = 0.194 *p* = 0.003) were significantly more satisfied with their GPs (F (3, 94) = 73.38, *p* < 0.001, corrected *R*^2^ = 0.691). Trust was significantly related (F (1, 96) = 72.861, *p* < 0.001, corrected *R*^2^ = 0.847) only to perceived competency (ß = .921, *p* < 0.001).

**Table 2:** Linear regression with backward selection for relationship-related factors

	Unstandardised coefficients		Standardised coefficient	t	*P*	95% confidence interval	
	b	SE	ß			lower	upper
Constant	0.271	0.221		1.230	0.222	−.167	0.710
Trust in GP	0.748	0.057	0.735	13.047	< 0.001	0.634	0.862
Talking about private topics	−0.042	0.021	−0.073	−1.969	0.052	−0.85	0.001
Satisfaction with GP	0.230	0.053	0.248	4.354	< 0.001	0.125	0.335
	Dependent variable: GP competency						
Constant	0.624	0.344		1.815	0.073	−0.059	1.301
Perceived GP competency	0.724	0.067	0.671	10.807	< 0.001	0.591	0.857
Showing emotions	0.087	0.037	0.150	2.324	0.022	0.013	0.161
GP asking about mental well-being	0.103	0.033	0.195	3.098	0.003	0.037	0.169
	Dependent variable: Satisfaction with GP						
Constant	0.572	0.221		2.590	0.011	0.134	1.010
Perceived GP competency	0.905	0.039	0.921	23.351	< 0.001	0.828	0.982
	Dependent variable: Trust in GP						

Variables included in step 1: trust in GP, GP competency, talking about private topics, showing emotions, GP asking about mental well-being, GP asking private questions, GP taking time to answer and satisfaction with GP

We performed an exploratory factor analysis to better understand the data. The Kaiser–Meyer–Olkin measure was 0.777, and Bartlett’s test of sphericity was significant with *p* < 0.001. The factor analysis revealed two underlying factors explaining 69.734% of the variance. Factor 1 included the variables Satisfaction (0.878), Competency (0.864), Trust (0.842), Time to Answer (0.684), Showing Emotions (0.658) and Well-Being (0.624). The second factor was made up of Private Topics (0.814) and Private Questions (0.788), indicating a split in the data between medical-oriented factors and factors relating to a more personal relationship between GPs and patients. The Supplementary Materials show the full analysis (Supplement Table S3).

### How important is the GPs’ opinion for patients?

**Fig. 2:**
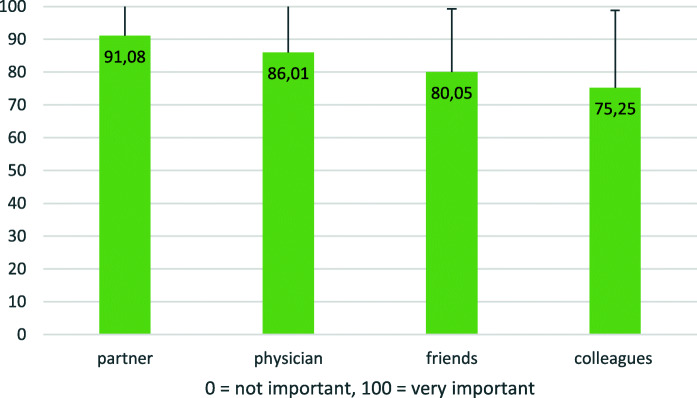
Mean value of importance of other people’s perception

To further understand the relationship between patients and GPs, we asked the patients to evaluate how important they perceive other people’s opinion of them. Analysis revealed that for the patients, what the GPs think about them was almost as important as what their own partners think. The perceptions of friends and former colleagues were, on average, less important to the patients (Fig. 2).

Then, we analysed which demographic and clinical factors are associated with a higher importance of the GPs’ opinions. For this purpose, we performed a block-wise linear regression with backward selection (F (3, 93) = 5.251; *p* = 0.002; corrected *R*^2^ = 0.117) using patient-related factors (block 1) and factors relating to the patient–GP relationship (block 2). The analysis is given in the Supplementary Materials (Supplement Table S4). Here, we found that the patients perceive GPs’ perceptions of them as more important if the GPs ask about their mental well-being (ß = 0.341; *p* = 0.002). However, patients who are single reported a reduced importance of GPs’ opinions (ß = − 0.205; *p* = 0.036).

### How do patients prepare for their visits to their GPs?

Among the patients, 65% reported making special preparations before GP consultations. Exploratory items revealed the nature of the patients’ preparation, which included bringing documents (*n* = 27), taking notes in advance (*n* = 26) or mentally preparing questions (*n* = 17), and personal hygiene (*n* = 16) (see Supplement Table S2). Then, we aimed to understand the reasons for these preparations using binary logistic regression with backward selection (Table 3). This model (F [10, 24] = 34.960, *p* < .001, *R*^2^ = 0.417) revealed that patients who perceived their GPs’ opinions of them as more important (ß = 1.032; *p* = 0.019) and who could show their emotions in front of the GP (ß = 1.471; *p* = 0.028) were more likely to prepare for a GP consultation. Furthermore, patients whose GP asks private questions (ß = 1.667, *p* = 0.04) tend to prepare more for consultations. However, frequent talking about private topics in general (ß = 0.59, *p* = 0.036) reduces the chances of patients preparing for consultations. Patients with stroke (ß = 0.066; *p* = 0.005) and Parkinson’s disease (ß = 0.122; *p* = 0.039) were less likely to prepare for GP consultations. Additional analyses using the Mann–Whitney U test and chi-square test are provided in the Supplementary Materials (Supplement Tables S5 and S6).

**Table 3:** Binary logistic regression with backward selection for preparation

	ß	SE	Wald	df	p.	Exp(ß)	95% confidence interval	
							lower	upper
Family status			6.500	2	0.039			
Family status (married)	0.267	1.434	0.035	1	0.852	1.306	0.079	21.715
Family status (widowed)	−1.301	1.462	0.792	1	0.373	0.272	0.015	4.778
GP competency	−0.770	0.412	3.498	1	0.061	0.463	0.207	1.038
**Talking about private topics**	**−0.527**	**0.252**	**4.385**	**1**	**0.036**	**0.590**	**0.361**	**0.967**
**Showing emotions**	**0.386**	**0.176**	**4.819**	**1**	**0.028**	**1.471**	**1.042**	**2.075**
**GP asking private questions**	**0.511**	**0.248**	**4.236**	**1**	**0.040**	**1.667**	**1.025**	**2.712**
**Importance of GP’s perception**	**0.032**	**0.013**	**5.515**	**1**	**0.019**	**1.032**	**1.005**	**1.060**
Diagnosis			9.502	3	0.023			
**Diagnosis (stroke)**	**−2.711**	**0.967**	**7.868**	**1**	**0.005**	**0.066**	**0.010**	**0.442**
**Diagnosis (PD)**	**−2.107**	**1.022**	**4.250**	**1**	**0.039**	**0.122**	**0.016**	**0.901**
Diagnosis (PNP)	−1.176	1.013	1.349	1	0.246	0.308	0.042	2.245
Constant	3.092	2.687	1.324	1	0.250	22.020		

Variables included in step 1: age, sex, family status, living situation, education level, employment status, frequency of GP consultations, duration of treatment, satisfaction with GP, GP competency, trust in GP, talking about private topics, showing emotions, GP asking about mental well-being, GP asking private questions, GP taking time to answer, importance of GP’s perception, total PHQ-9 score and diagnosis

### How do patients want to be perceived by their GPs?

Exploratory data were collected on the patients’ beliefs of GPs’ current perceptions of them and how they wanted to be perceived (Desired Perception) to understand the motivation for patient behaviours (see Supplement Table S2). Inter-rater reliability for 3 raters measured by Fleiss Kappa was substantial both for current (κ = 0.945, *p* < 0.001) and desired perception (κ = 0 .846, *p* < 0.001). The topics patients mentioned most in the exploratory items were wanting to be perceived as engaged, cooperative and actively participating (current perception *n* = 30, desired perception *n* = 19) as well as being pleasant (current perception *n* = 29, desired perception *n* = 26). Regarding their desired perception, several patients stressed wanting to be seen as an individual, respected person (*n* = 29).

## Discussion

In this study, we aimed to understand factors relating to the expectations and behaviour of neurogeriatric patients and their relationships with their GPs.

Overall, the patients reported a high frequency of monthly consultations and long treatment duration, which may also be due to the patients’ age and the chronicity of their disorders. Furthermore, the patients in this study claimed high treatment activity where they rarely cancel appointments, use pre-screenings and prepare for consultations. This is at odds with the literature indicating that at least half of the patients are nonadherent [8, 13, 14]. A possible reason for this is that patients reported high levels of satisfaction and trust, which are known to enhance adherence [8, 53]. In the next step, we aimed to understand to which factors these high levels of trust and satisfaction are attributed.

Generally, the current data describe two distinct aspects of GP consultations for neurogeriatric patients. There is a certain social component, such as talking about private topics, which was mentioned to be important by some patients. However, the data show a split in the presented patient group regarding social aspects, with only approximately a third of the patients talking about private matters with their GPs. Instead, the focus of our patients lies on the medical aspects. This split in the data is further supported by an exploratory factor analysis revealing two factors, one containing items regarding the social component, such as talking about private topics, and the other encompassing medical aspects such as GP competence and trust. Interestingly, a subcomponent relating to empathetic psychosocial aspects, such as showing emotions and talking about mental well-being [54, 55], was linked to medical rather than social aspects. This conforms to other studies reporting two functional needs of patients in GP consultations, encompassing both a private relationship and the transfer of medical information [20]. For our group of chronically ill patients, the common notion of ‘older people see their GPs just to have someone to talk to’ seems to be overruled by the need to be cared for medically and emotionally. This is in line with other research highlighting the almost therapeutic role of GPs for their elderly patients that goes beyond simple social interaction [2]. Accordingly, the patients’ expectations for GP consultations derived from the exploratory items put a focus on medical aspects, such as health screenings, prescriptions and referrals.

Analysis revealed that the three factors Competency, Satisfaction and Trust in GP are closely related, indicating that satisfaction with and especially trust in GP are primarily linked to medical expertise [56]. Interestingly, a frequent talking about private topics reduced perceived GP competency, again indicating that patients tend to prioritise medical aspects. This is further supported by other studies linking patient satisfaction to discussions on physical health rather than social factors [57].

Having a GP who asks about the patient’s mental well-being and the ability to show emotions in front of the GP were further related to satisfaction with the GP. This psychosocial aspect to the relationship may reflect properties of the specific group of chronically ill older people, for whom deterioration of mental well-being plays a key role [2, 58]. Older patients seek a deeper connection based on caring and emotional support from their GPs [32]. The chronic nature of illnesses reported in this study may be accompanied with higher emotionality and may add an integral psychosocial component to the relationship [38]. Likewise, previous research has shown that a focus solely on biomedical topics leads to reduced satisfaction compared to focussing on psychosocial topics, again highlighting the important role of mental well-being for this particular patient group [46]. Additionally, patients frequently expressed the desire to be taken seriously, to be listened to and to have time for questions [22, 38, 59], which serves as a therapeutic and caring strategy and strengthens the relationship between GPs and patients [2, 60].

Furthermore, the patients attributed a high value to their GPs’ perceptions of them, comparable to those of their partners. This importance patients placed on their GPs’ opinions of them is significantly linked to being able to speak about mental well-being, presenting further evidence that patients value psychosocial care [54, 61]. The role of an empathic, caring and understanding GP has been previously highlighted [38, 62, 63], citing a focus on the patients’ personal experiences as an important factor in healthcare. Empathetic care reduces the barrier of not wanting to be bothersome because patients feel that their GPs are interested in their well-being [22, 38, 59]. The reduction of this barrier is especially important for elderly patients, who are used to a biomedical model of healthcare in which the doctor makes the decisions and the patient complies without being able to communicate their needs [2, 45]. Additionally, the burden of multiple health problems may lead to forgetting of certain aspects and uncertainty about which issues to raise, especially under time pressure. This risk can be reduced with an appropriate patient-GP-relationship [49].

In addition to feeling taken care of, this empathetic approach may allow patients to participate more actively [64]. This is supported by our results, because patients who could show emotions in front of their GPs were more likely to prepare for consultations. Analysis regarding patient behaviours revealed that 65% of the patients prepared for GP consultations, which conforms to the findings of other studies showing that the better part of patient participation comes from the patients themselves rather than being initiated by the GP [50, 59]. A closer look at the factors that contributed to this patient-initiated behavior revealed that, in addition to empathy, preparation levels were higher in patients who allocated a high level of importance to their GPs’ perceptions of them. This implies that patients who place value on their GPs’ opinions want to be perceived as active and engaged. This idea is further supported by the analysis of the exploratory data regarding GP perception. For most patients, being perceived as likable is important, which is at odds with the literature often citing and focusing on patients with difficult behaviors [34–36]. Unlike those studies, our data revealed that most patients put in effort, which may again be based on the patients’ high dependency on their GP. Accordingly, our data suggest that this desire to be perceived as pleasant does not derive from a simple need for social interaction but from the need for medical care; patients might fear negative consequences regarding their healthcare when perceived as difficult and thus might put in more effort in the hopes of benefiting from a good GP relationship [45, 65].

It is important to foster this effort, as the literature indicates that patient preparation leads to a more active patient participation [18] and better knowledge after the consultation [66], because preparation helps patients prioritise and remember their needs [64]. Preparation further aids the GP’s decision-making as it enables patients to ask questions and express their opinions and concerns [16]. Thus, patient preparation leads to an overall more effective consultation.

In contrast to psychosocial care, frequent talking about private topics reduces the chances of preparation, indicating that patients did not place as much weight on the medical aspects of the consultation and thus would not feel the need to prepare. This finding again suggests that the patients did not simply seek social interaction from GP consultations [2].

In addition, diagnoses of stroke and Parkinson’s disease were associated with a reduction in preparation, indicating that different patients have different needs depending on diagnosis [24]. However, analysing the exact nature of expectations for even smaller subgroups of patients is out of the scope of this study.

As an overall conclusion, older patients with chronic illness place high value on their GPs. This importance stems from the need to be cared for medically and emotionally rather than socially and leads to a better patient preparation for GP consultations, resulting in more efficient consultations. When provided with an empathetic environment and medically competent care, patients are willing to participate actively and prepare for consultation. This finding sheds further light on older patients’ motivation regarding their own health, indicating that the often discussed nonadherence does not necessarily come from the patients’ purposeful choices but may reflect either difficulties in understanding medical information or barriers hampering open communication, such as lack of empathetic understanding [67]. Thus, a specific form of communication is needed when dealing with older adults to ensure satisfaction and best healthcare outcomes [13, 14]. This is especially important as the discrepancy of patients’ current and desired perception in this study indicates that older adults do not feel appropriately treated by their GPs. This conforms to the results of the study by Kojer [68] stating that GPs fail to recognise their older patients as individual people, even though high-quality care is associated with a respectful whole-person approach that is emotionally supportive [69].

Patients’ expectations regarding GP consultations encompass two closely related key aspects, including medical competence as well as emotionally caring, empathetic listening and understanding. Both factors are important to foster trust and satisfaction in patients, and psychosocial care encourages patients to take an active role in their healthcare. Older persons in particular constitute a highly vulnerable group of patients, for whom the frequent GP consultations serve not only as opportunities to improve their physical health but also to speak about emotional well-being. Thus, the GP-patient relationship can go much further than the biomedical model of health and should also incorporate emotional care to improve health outcomes. Especially for elderly patients who may struggle with handling multiple conditions, a declining mental health, a shrinking social circle and complex medication plans, a supportive relationship with their GPs can become a valuable resource that should not be underestimated in its influence [2]. Our results indicate that older adults are willing to put in effort to receive due care, thus an appropriate setting should be provided in the medical context to allow for effective consultations. However, expectations vary between patients, and even in a relatively specific patient group, the needs of some subgroups of patients are not yet fully understood.

### Limitations and future research

A clear limitation of this study is its cross-sectional and exploratory design, which does not allow for any interpretation of causality.

Due to the exploratory design and focus on the open questions, most variables such as Trust and Satisfaction were assessed with single item measures. Thus, these items reflect the general, broad assessment of patients’ overall trust and satisfaction, but cannot map out the full, complex nature of these constructs. While our data allow a first insight into the role of these constructs for the patient-GP-relationship, in-depth studies using validated questionnaires are needed to confirm those results.

Furthermore, the current data reflect the needs and experiences of a specific group of patients, that is, chronically ill older adults with neurological disorders. It is possible that these patients were highly dependent on their GPs due to the nature of their illnesses and thus place much value on their relationship with their GP. The surprising finding that treatment duration and frequency of GP consultations hardly play a role in this patient group may be because the medical necessity for these consultations effectively precludes any effects of personal motivation. Thus, the generalisability of the results is limited.

As specific groups of patients seem to vary in their needs and expectations [24], future studies might be able to shed light on the exact factors relating to specific needs of different patient groups. The results of this study serve as suggestions for important components in the GP–patient relationship; however, proposing exact methods on how to implement those components into the healthcare system is out of the scope of this study. Especially with regard to the influence on patient adherence and improved health outcomes, establishing GP relationships in which older patients feel cared for is a thus far underestimated but highly important resource in healthcare [2].

## Supplementary Information

**Additional file 1: Table S1.** Translation of the questionnaire. **Table S2.** Exploratory general practitioner (GP)-related patient behaviour. **Table S3.** Exploratory factor analysis for relationship-related factors. **Table S4.** Block-wise linear regression with backward selection for importance of physician’s perception. **Table S5.** Mann-Whitney U test for people with and without preparation for consultation. **Table S6.** Chi^2^ Test/Fishers Exact Test for people with and without preparation for consultation.

## Data Availability

Dataset is available upon request from the corresponding author.
