# Supplementary material for: Expectations and behaviour of older adults with neurological disorders regarding general practitioner consultations: an observational study
Source: BMC Geriatr. 2021 Sep 25;21:512. doi: 10.1186/s12877-021-02469-3 (PMC8466933; doi:10.1186/s12877-021-02469-3)
Supplement: Supplementary file 1 — Additional file 1: Table S1. Translation of the questionnaire. Table S2. Exploratory general practitioner (GP)-related patient behaviour. Table S3. Exploratory factor analysis for relationship-related factors. Table S4. Block-wise linear regression with backward selection for importance of physician’s perception. Table S5. Mann-Whitney U test for people with and without preparation for consultation. Table S6. Chi2 Test/Fishers Exact Test for people with and without preparation for consultation. [file 12877_2021_2469_MOESM1_ESM.docx]

**Table S1.** Translation of questionnaire.

General Practitioner (GP)-Related Patient Behaviour

1. Do you arrive on schedule for your GP consultation?

If yes, why?

If no, why not?

1. Do you make any preparations for your GP consultation? (e.g. taking medical records with you, looking up your last test results)
2. Do you allow for sufficient time for your GP consultation?
3. Which additional preparations do you make for your GP consultation? Do you have any routines before your GP consultations? Have you ever done anything out of the norm to prepare for your GP consultation? If yes, what?
4. How frequently do you consult your GP? (quarterly)
5. Do you go to additional preventive medical check-ups? How often?
6. Do you additionally consult your GP when you are feeling worse, or do you wait until your next scheduled appointment?
7. Do you cancel GP consultations, e.g., due to private reasons?
8. How many weeks in advance do you schedule new GP consultations?

GP-Patient Relationship

1. How long have you been a patient at this GP practice (in years)?
2. What were your reasons for choosing this GP?
3. How would you describe your tone of communication with your GP? (e.g. friendly, cordial, reserved)
4. How would you describe your GP’s tone of communication with you? (e.g. friendly, cordial, reserved)
5. Are you satisfied with your GP?

Always Mostly Often Sometimes Rarely Never

1. Do you perceive your GP as competent and qualified?

Always Mostly Often Sometimes Rarely Never

1. Do you trust your GP?

Always Mostly Often Sometimes Rarely Never

1. Do you speak about private matters with your GP?

Always Mostly Often Sometimes Rarely Never

1. Can you show your emotions in front of your GP? (give example in case patients struggle to respond, such as crying in front of your GP)

Always Mostly Often Sometimes Rarely Never

1. Does your GP inquire about your mental well-being on top of your physical health?

Always Mostly Often Sometimes Rarely Never

1. Does your GP ask private questions?

Always Mostly Often Sometimes Rarely Never

1. Does your GP take sufficient time to listen to you and reply to your questions?

Always Mostly Often Sometimes Rarely Never

1. Have you ever talked negatively about another person behind their back?

Always Mostly Often Sometimes Rarely Never

Patient Expectations

1. Which of the following expectations do you have for a GP consultation?
   - 1. Social contact
     2. Check-ups
     3. Assessing the current state of health
     4. Prescription of medication
     5. Other prescriptions, e.g. physiotherapy
     6. Planning the process of your medical care
     7. Monitoring the process of your medical care
     8. Other (please explain)

Importance of GP perception

1. How do you believe your GP currently perceives you?
2. On a scale of 0 = not important at all to 100 = extremely important, how important is your partner/colleagues/friends/GP’s perception of you?
3. If there are differences in the importance of perception, why?
4. How would you like to be perceived by your GP?

**Table S2.** Exploratory general practitioner **(**GP)-related patient behaviour.

|  |  | **n** |
| --- | --- | --- |
| Reason for choosing GP | Practice takeover | 36 |
|  | Proximity | 26 |
|  | Recommendation by others | 13 |
|  | Family members at same practice | 5 |
|  | Lack of alternatives | 5 |
|  | Impaired mobility | 3 |
|  | Other | 17 |
| Punctuality | Yes | 98 |
|  | No | 2 |
| Cancelling of GP appointments for private reasons | Yes | 7 |
|  | In exceptional cases | 14 |
|  | No | 79 |
| Additional consultation when feeling worse | Yes | 88 |
|  | No | 12 |
| Additional preventive check-ups | Yes | 86 |
|  | No | 14 |
| Kinds of check-ups used | Dentist | 56 |
|  | Ophthalmologists | 32 |
|  | Urologists | 22 |
|  | Gynaecologists | 13 |
| Expectations for GP consultations | Prescription of medication | 86 |
|  | Health Screening | 84 |
|  | Other prescriptions | 75 |
|  | Referral to specialists | 49 |
|  | Monitoring of treatment course | 36 |
|  | Planning of treatment course | 34 |
|  | Social contact | 25 |
|  | Other, i.e. listening, advice, answering questions, getting immediate help | 14 |
| Preparation | Bringing documents, looking at latest test results | 27 |
|  | Taking notes in advance about questions and procedures, doing research | 26 |
|  | Mentally preparing questions | 17 |
|  | Personal hygiene | 16 |
|  | Measuring blood pressure and glucose level | 4 |
| Current Perception  Engaged | **Patient responses**  Engaged, cooperative, making an effort, prepared, participating, informed | 30 |
| Pleasant | Friendly, nice, open-minded, warm, hearty, agreeable, approachable | 29 |
| Ill | Ill, obese, GP knows patient has problems, GP sees it as it is, chronic problems | 16 |
| As a Patient | As a patient, as a customer, as someone who needs a doctor | 11 |
| Normal | As a normal person | 9 |
| Resilient | Normal despite handicap, strong, has been through a lot, light-hearted, respectable, positive, resolute, resistant | 8 |
| Healthy | Healthy, fit, healthy for patient’s age | 8 |
| As an individual | Valuable, respected | 3 |
| Desired Perception | **Patient responses** |  |
| Engaged | Cooperative, informed, organised, prepared, engaged, involved, educated, knowledgeable | 19 |
| Pleasant | Approachable, open-minded, fair, nice, warm, friendly, funny, respectful, leaving a good impression | 26 |
| As a patient | As a normal patient, as a patient | 6 |
| Normal | Normal, not ‘extra’ | 17 |
| Resilient | Strong, resistant, positive, fighting, light-hearted despite difficult situation | 4 |
| As an individual | As human, as a whole, wants to be seen, as an individual, equal, valuable, wants to be taken seriously, wants to be respected | 29 |

**Table S3**. Exploratory factor analysis for relationship-related factors.

|  | Component loadings | |
| --- | --- | --- |
|  | 1 | 2 |
| Satisfaction with physician | .878 | -.182 |
| Physician competency | .854 | -.395 |
| Trust in physician | .842 | -.376 |
| Talking about private topics | .333 | .814 |
| Showing emotions | .658 | .209 |
| Physician asking about mental well-being | .624 | .361 |
| Physician asking private questions | .387 | .788 |
| Physician taking time to answer | .684 | -.183 |
| Eigen Values for Component | 3.420 | 2.159 |
| Variance explained (%) | 42.748 | 26.986 |
| Extraction Method: Principal Component Analysis, Rotation Method: [Varimax](https://www.sciencedirect.com/topics/medicine-and-dentistry/varimax-rotation) with Kaiser Normalization | | |

| **Table S4.** block-wise linear regression with backward selection for importance of physician perception | | | | | | | | |  |
| --- | --- | --- | --- | --- | --- | --- | --- | --- | --- |
| Model | | Unstandardized coefficients | | Standardized coefficient | t | p | 95% confidence interval | |  |
|  |  | b | SE | ß |  |  | lower | upper | Corrected R² |
|  | (Constant) | 81.370 | 4.320 |  | 18.837 | .000 | 72.792 | 89.949 | .117 |
|  | Family Status Single | -20.198 | 9.469 | -.205 | -2.133 | .036 | -39.002 | -1.394 |  |
|  | physician asking about mental well-being | 3.412 | 1.052 | .341 | 3.242 | .002 | 1.322 | 5.502 |  |
|  | Physician asking private questions | -2.375 | 1.244 | -.201 | -1.908 | .059 | -4.846 | .097 |  |
| dependent variable: importance of physician's perception. Variables included in block 1: age, sex, family status, living situation, education level, employment status, PHQ-9 score, diagnosis; block 2: frequency of GP consultations, duration of treatment, satisfaction with GP, GP competency, trust in GP, talking about private topics, showing emotions, GP asking about mental well-being, GP asking private questions, GP taking time to answer, importance of GP's perception. | | | | | | | | |  |

**Table S5.** Mann-Whitney U test for people with and without preparation for consultation.

|  | Mann-Whitney U Test | Wilcoxon W | Z |  | *p* |
| --- | --- | --- | --- | --- | --- |
| Age | 1025.000 | 1655.000 | -.814 |  | .418 |
| Frequency of physician consultation | 1014.000 | 1609.000 | -.575 |  | .569 |
| Duration of treatment | 1087.500 | 3232.500 | -.364 |  | .719 |
| Satisfaction with physician | 1098.500 | 1728.500 | -.332 |  | .745 |
| Physician competency | 1018.500 | 3163.500 | -1.211 |  | .239 |
| Trust in physician | 1115.500 | 3260.500 | -.238 |  | .821 |
| Talking about private topics | 1127.500 | 3272.500 | -.074 |  | .943 |
| Showing emotions | 855.000 | 1485.000 | -2.103 |  | .035 |
| Physician asking about mental well-being | 1045.000 | 1738.500 | -.086 |  | .933 |
| Physician asking private questions | 1045.000 | 1675.000 | -.688 |  | .495 |
| Physician taking time to answer | 1091.500 | 1721.500 | -.452 |  | .678 |
| Importance of physician’s perception | 914.500 | 1544.500 | -1.565 |  | .118 |
| PHQ* | 983.500 | 1613.500 | -1.120 |  | .265 |

*Patient-Health Questionnaire-9 to assess depression.

**Table S6.** Chi² Test/Fishers Exact Test for people with and without preparation for consultation.

|  |  | Value | Df | Asymptotic Sign. | Exact sig. |
| --- | --- | --- | --- | --- | --- |
| Sex | Pearson Chi² | .457 | 1 | .499 | .532 |
| Diagnosis | Pearson Chi² | 12.307 | 3 | .006 | .005 |
| Family Status | Fisher’s Exact Test | 7.647 |  |  | .016 |
| Living Situation | Pearson Chi² | 2.811 | 1 | .094 | .104 |
| Education | Pearson Chi² | 1.775 | 2 | .412 | .435 |
| Employment Status | Fisher’s Exact Test | .753 |  |  | 1.00 |
